# Differences in Body Composition, Muscle Strength, and Power Between Young Karate Athletes of Different Competing Disciplines: A Pilot Study

**DOI:** 10.3390/life16050801

**Published:** 2026-05-11

**Authors:** Bojan Pavlović, Vanja Cicović, Ljubica Lalović, Borislav Cicović, Lazar Toskić, Nikola Aksović, Veroljub Stanković, Ljubiša Lilić, Marko Tomić

**Affiliations:** 1 Faculty of Physical Education and Sports, University of East Sarajevo, 71126 Lukavica, Bosnia and Herzegovina; bojan.pavlovic@ffvis.ues.rs.ba (B.P.); vanja.cicovic@ffvis.ues.rs.ba (V.C.); ljubica.milanovic@ffvis.ues.rs.ba (Lj.L.); borislav.cicovic@ffvis.ues.rs.ba (B.C.); 2 Faculty of Sport and Physical Education, University of Priština in Kosovska Mitrovica, 38218 Leposavić, Serbia; lazar.toskic@pr.ac.rs (L.T.); veroljub.stankovic@pr.ac.rs (V.S.); ljubisa.lilic@pr.ac.rs (Lj.L.); markotkarateks@gmail.com (M.T.); 3 Faculty of Sport, University “Union–Nikola Tesla”, 11070 Belgrade, Serbia

**Keywords:** martial art, kata, kumite, young karatekas, body composition, muscle strength and power

## Abstract

(1) Background: This study aims to investigate the differences in body composition, muscle strength, and power between young karate athletes from different competitive disciplines. (2) Methods: The study sample consisted of 131 participants (69 boys and 62 girls) aged 10–14 years divided into three groups: kata (n = 48), kumite (n = 40), and athletes competing in both disciplines (n = 43). The set of variables included 1 anthropometric variable, 6 variables for assessing body composition (InBody 270), 8 variables for assessing handgrip strength (handgrip strength test, Fmax, RFD, absolute and relative, both hands), and 3 variables for assessing lower limb muscle power (force plate, CMJ height and power). Of the statistic analysis, MANOVA and ANOVA, along with MANCOVA and ANCOVA were performed. (3) Results: The adjusted results revealed significant general differences between groups (from *p* = 0.005 to *p* = 0.009). Regarding body composition, kumite athletes are taller, have greater body mass, body water, proteins, minerals, and muscle mass content than kata athletes and athletes specialized in both disciplines (from *p* = 0.002 to *p* = 0.045). The young karate athletes specialized in kumite competition exhibit higher levels of absolute handgrip muscle strength, rate of force development, and absolute lower limb muscle power than kata athletes and athletes specialized in both disciplines (from *p* = 0.002 to *p* = 0.041). There were no significant differences in any measured parameters between kata athletes and young karate athletes specialised in both disciplines. (4) Conclusions: The results are associated with higher values of body composition, muscle strength, and power in kumite athletes compared to kata athletes and athletes competing in both disciplines.

## 1. Introduction

Martial arts sports belong to a group of disciplines characterized by diverse adaptations reflected in the evolution of competitive activity, training methodologies, and scientific investigation [1,2,3]. Karate is defined from multiple perspectives, but in modern sport it is primarily regarded as a system of self-defence and a combat sport with specific physical and technical demands [4,5,6].

Karate has evolved into a modern sport discipline and is now one of the most widely practiced sports. It comprises two competitive categories: kata (forms) and kumite (sparring) [7,8,9]. Changes in judging rules, particularly in sport fighting, have led to differences in training and competitive demands between these disciplines. Consequently, specialization has increased, and it is now uncommon for athletes to compete in both disciplines [10,11].

Kata consists of defined sequences of offensive and defensive techniques. These techniques are highly formalized, performed in a strictly predetermined order, sometimes relatively slowly and in relatively low stances. In addition to strikes and blocks, kata may also include lunges, jumps, turns, and sliding movements. It is defined as an imagined fight with an opponent, which, in essence, it is—albeit in a technically richer and more complex form [12,13]. On the other hand, kumite is defined as a sport discipline involving poly-structural, acyclical movements aimed at the symbolic defeat of an opponent. It requires a high level of technical skills and fine motor control under both static and dynamic conditions, accompanied by the ability to perform dominant technical actions (strikes, blocks, feints, clearings, and movements) in the shortest possible time. Kumite consists of freely chosen offensive and defensive actions directed at the opponent, forming a sequence of interconnected techniques and movements that require continuous body displacement in space [14,15]. Most of the technical elements that comprise kata are not applied in kumite, making kata structurally a more demanding discipline. Furthermore, practicing kata can be very effectively used to improve kumite techniques, and it is also believed that regular kata training enhances overall and explosive body strength, as well as balance and agility. Based on the above, it can be concluded that kumite exhibits characteristics of an open motor pattern, involving direct contact with an opponent, whereas kata falls under closed motor pattern activities, consisting of schematized movements without direct contact with an opponent [8,10,11].

The existence of distinct training programs for kata and kumite is based on the differences between these disciplines. Training in karate for school-aged children, as in other sports, differs in applied methods and means compared to training older age groups. At this age, greater attention is paid to technical preparation, with training focused, through various methodological approaches, on the acquisition of basic karate techniques. Technical preparation begins with instruction and continues through training until movement habits are fully acquired [13]. However, in order for karate techniques to be performed correctly and efficiently, a certain level of motor abilities is required [16]. Research has shown that systematic physical training in karate can positively influence the development of physical fitness and body composition [4,16,17,18]. Exercises involved in learning karate techniques activate the entire musculature and develop both the left and right sides of the body equally, thereby avoiding the unilateralism observed in some other sports. More precisely, the dominant feature of karate techniques consists of fast, explosive movements with an emphasis on controlled application of force against the opponent [5].

Karate is one of the most widespread martial arts in the world, where elite performance and sports results depend on a high level of motor and functional abilities, appropriate body composition and anthropometric characteristics, technical execution, and mental stability [4,9,18,19]. This is particularly relevant to muscle contractile characteristics and body composition, which undoubtedly represent crucial factors significantly influencing the sports performance of karate athletes. Muscle contractile characteristics, such as muscle strength and power, being among the most important abilities enabling movement, likely assume a crucial role in all sports activities [20]. Furthermore, body composition, that is, the proportion of muscle, fat, water, and minerals in the body, affects overall health and, consequently, has a substantial impact on success in almost all sports [21]. Accordingly, karate, as a physically and structurally complex sport, largely depends on both muscle strength and power [22] and body composition [18]. Moreover, karate is a sport with a distinctive competitive aspect, as competitions are conducted in different movement and competitive formats, namely kata and kumite. In this context, the selected variables reflect key physical demands of karate disciplines. Handgrip strength and rate of force development (RFD) represent upper-limb force production and rapid force generation, which are relevant for fast and controlled striking actions, particularly in kumite [20,22]. Countermovement jump (CMJ) height and power reflect lower-limb explosive capabilities, important for kicking, rapid displacement, and changes in direction [5,8]. Although kata emphasizes technical precision and control, adequate levels of strength and power are still required, suggesting that these variables may differ in their contribution across disciplines.

Examining differences in body composition, muscle strength, and power among karate athletes of different disciplines is crucial for coaches, strength and conditioning specialists, sports scientists, physiotherapists, and the athletes themselves, as this knowledge can inform targeted and effective training strategies. This is particularly important for young karate athletes, for whom every correctly or incorrectly applied intervention can have significant consequences for their health, proper growth and development, as well as achieving elite sports performance. However, previous studies have examined differences between kata and kumite primarily in adult karate athletes [9,10,23], indicating that most existing evidence is based on adult populations, while research in younger athletes remains limited. These between-group differences may be explained by two complementary mechanisms: training-induced adaptations arising from the specific demands of kata and kumite training, and selection or morphological predisposition, whereby athletes with certain physical characteristics are more likely to specialize in a given discipline [5,9]. Nevertheless, no study has investigated or compared body composition, muscle strength and power among young karate athletes across different competitive disciplines.

Therefore, this study aims to investigate the differences in body composition, muscle strength, and power between young karate athletes from different competitive disciplines. The study specifically focuses on athletes specializing in kata, kumite, and karate athletes specialized in both disciplines. Based on the defined aim, it was hypothesized that differences in body composition, muscle strength, and power would be observed among young karate athletes from different competitive disciplines.

## 2. Materials and Methods

A cross-sectional study design was employed; therefore, causality between variables cannot be inferred. Participants were categorized into three groups according to their competitive specialization: kata, kumite, and athletes who compete in both disciplines. The study was conducted as a pilot investigation.

### 2.1. Sample of Participants

The study sample consisted of 131 participants (69 boys and 62 girls) aged 10–14 years. Participants were recruited from multiple karate clubs across different cities during a national karate training camp, which served as the data collection setting. The sample was divided into three groups according to the athletes’ primary competition discipline based on their competition participation during the season: kata (n = 48), kumite (n = 40), and athletes competing in both disciplines (n = 43). All participants practiced Shotokan karate, had at least two years of training experience, and competed at national and international levels within their respective age categories. Biological maturation data were not collected in this study. Detailed descriptive characteristics of the participants are presented in Table 1. Inclusion and exclusion criteria were established to ensure sample homogeneity and the relevance of the collected data.

Inclusion criteria:

- Minimum of two years of active karate training;
- Active participation in competitions within their age category;
- Age within the defined range (10–14 years);
- Voluntary participation with written consent from parents/guardians for minors;
- Registration in a karate club recognized by the national federation;
- Regular participation in competitions during the previous season.

Exclusion criteria:

- Injuries or medical conditions affecting body composition, muscle strength and power, or performance;
- Recent surgery or prolonged break from training (>1 month);
- Irregular attendance at training sessions;
- Lack of written consent for participation;
- Participation in other sports at a competitive level that may affect muscle strength and power, as well as body composition;
- Use of medications or supplements that may influence muscle mass or performance.

All participants provided written informed consent to take part in the study and were free to withdraw from the experimental programs at any time. The study was conducted in accordance with the principles of the Declaration of Helsinki and received approval from the Ethics Committee of the Faculty of Physical Education and Sports (IRB: 484-2, 3 July 2025).

### 2.2. Sample of Variables

The set of variables included 1 anthropometric variable (body height), 6 variables for assessing body composition, 8 variables for evaluating handgrip strength (upper-limb isometric strength), and 3 variables for assessing lower limb muscle power (Table 2).

### 2.3. Measurement Procedures

The measurements were conducted in the morning hours under standardized conditions. All participants underwent an identical testing protocol designed to minimize external influences and ensure the reliability of the collected data. Body composition was assessed first, in accordance with the manufacturer’s recommendations. This was followed by a structured 15 min warm-up protocol (running, jumping, squats, stretching) intended to prepare the participants for the subsequent assessments of muscle strength and power. After the warm-up, the muscle strength and power tests were performed in a predetermined order to avoid fatigue-related effects that could influence the results.

Prior to testing, all participants were thoroughly informed about the purpose of the study, the measurement procedures, and the demands of each test. They were advised to refrain from intensive physical activity for at least 24 h before the measurements and to maintain their usual hydration and dietary habits. The measurements were carried out in cooperation with the Faculty of Sport and Physical Education. To ensure procedural consistency and minimize inter-rater variability, all assessments were conducted by the same experts, coaches, and authors of the study.

#### 2.3.1. Body Composition Measurement Procedures

Body composition was assessed using the InBody 270 device (Biospace Co., Ltd., Seoul, Republic of Korea). All measurements were carried out according to the manufacturer’s guidelines and aligned with protocols used in previous studies [21,24]:

- Measurements were performed in the morning hours, from 8:00 to 10:00 a.m.;
- Participants were requested to refrain from heavy meals after 9:00 p.m. the night before the assessment and to abstain from any eating or drinking before the measurements on the testing day;
- Participants were instructed to avoid intense physical activity for 24 h before the measurements, ensuring that their last training session was completed at least 12 h prior to testing;
- Participants abstained from alcohol for 48 h and refrained from consuming diuretics (e.g., coffee, chocolate) prior to the measurements;
- Participants emptied their bladders before the measurements;
- Participants stood in an upright position for 5 min prior to the measurements;
- The measurements were performed with participants in a standing position, as recommended by the manufacturer, with their arms positioned alongside the body and approximately 15 cm away from the torso.

During testing, the participants wore only underwear, and they were instructed to remove all metallic jewellery and other metal objects. Prior to testing, all participants were informed about the measurement procedure. They were first asked to step onto the scale and place their hands and feet on the designated contact surfaces. During the measurement, participants were required to stand upright, remain still, and keep their bodies relaxed—that is, without tensing the arms, legs, or torso—to look straight ahead, breathe normally, and refrain from speaking. The measurement lasted approximately two minutes. All measurements were performed under identical conditions, and all participants were healthy and complied with the required pre-measurement procedures. Additionally, prior to the measurement, the participants’ body height was assessed using a standard anthropometer (GMP instruments, Susten, Switzerland) with an accuracy of 0.01 cm.

#### 2.3.2. Handgrip Strength Measurement Procedures

Handgrip strength was assessed using isometric dynamometry, employing a hand dynamometer (Sports Medical Solutions, Belgrade, Serbia) and the handgrip test. The device was calibrated according to the manufacturer’s guidelines prior to testing, and the signal was recorded at a sampling frequency of 500 Hz. The measurement procedure was conducted in accordance with previous studies [25,26]:

- The participant sits on a chair with their back straight, without leaning;
- The participant holds the hand dynamometer with a full grip in the hand, with the elbow fully extended alongside the body;
- On the examiner’s signal, the participant performs a maximal and rapid contraction of the hand flexor muscles, squeezing the dynamometer as hard and fast as possible.

Prior to the measurements, participants were familiarized with the testing procedure and performed several practice trials. The testing procedure was standardized and performed in a consistent manner across all participants, with familiarization trials conducted prior to testing. Maximal voluntary contractions were executed as brief, maximal and rapid efforts, in a duration of approximately 3 s (until the maximal value of the muscle was exhibited), and in accordance with previously described protocols to ensure test reliability and reproducibility [25]. The hand grip width was set to correspond with participants’ age, that is, hand length. Hand dominance was determined based on self-reported preference for writing and preferred striking or kicking limb in daily sport-specific activities. Measurements were conducted for both hands, with the results reported separately for the right and left hands. A 3 min rest period was used between trials to minimize fatigue [25]. The highest value from the two trials was recorded as the final result. RFD was calculated from the force–time curve as the slope of force increase from contraction onset to peak force (Δforce/Δtime, 50–80% of maximal force). The highest RFD value obtained across trials was used for analysis. Raw force signals were processed using the manufacturer’s proprietary software (SMS) without additional filtering. Additionally, relative values of maximal force and RFD were obtained, according to BW [27].

#### 2.3.3. Muscle Power Measurement Procedures

Muscle power was assessed using isotonic dynamometry, employing force platforms (K-Deltas, Kinvent, Montpellier, France) and the Countermovement Jump (CMJ) test. The force platform system recorded data at a sampling frequency of 1000 Hz. The device was automatically calibrated before testing.

The measurement testing procedure was carried out in accordance with the guidelines established in previous studies [28,29]:

- The participant stands on the platform at a marked position, with feet placed parallel and hip-width apart;
- Hands are kept at the sides;
- From this position, the participant performs a downward movement followed by a rapid upward extension, extending all lower-limb joints and jumping vertically, while keeping the hands at the sides throughout;
- The jump is performed three times;
- Participants are instructed to jump maximally, avoid excessive or additional knee flexion beyond the standard countermovement and avoid any preparatory or bouncing movements prior to the jump.

Trials in which participants lost balance, removed their hands from the hips, or did not perform a clear countermovement were considered invalid and repeated. Before the assessment of muscle power, participants were familiarized with the test, performed practice trials, and completed a warm-up. The testing procedure was performed in a standardized manner across all participants to ensure consistency and reliability. The maximum values of power and jump height from the three trials were recorded as the final results. A 1 min rest interval was provided between trials to minimize fatigue. Jump height and power were automatically calculated by the force platform software (Kinvent application, version 2.23.00) based on force–time characteristics [28,29]. Additionally, relative values of muscle power were obtained, according to BW [30].

### 2.4. Statistical Analysis

Data were analyzed using the statistical software SPSS (version 25.0, SPSS Inc., Chicago, IL, USA). For all measured variables, descriptive statistics were calculated, including the mean and standard deviation (SD). To investigate differences between kata, kumite, and athletes competing in both disciplines, multivariate analysis of variance (MANOVA) and univariate analysis of variance (ANOVA) were performed. Preliminary analyses indicated that the assumptions of normality (Kolmogorov–Smirnov test), linearity, homogeneity of variances, and homogeneity of regression slopes were satisfied. In addition, multivariate analysis of covariance (MANCOVA) and analysis of covariance (ANCOVA) were performed to control for the effects of sex, age, and body height, with Bonferroni post hoc tests applied to adjust for multiple comparisons. The sample included a total of 131 participants, which was considered sufficient to perform the applied statistical analyses and to detect significant differences, in accordance with accepted guidelines [31]. The level of statistical significance was set at *p* < 0.05.

## 3. Results

The results of the MANOVA analysis (Table 3) showed significant multivariate effects for competitive discipline on both body composition (Wilks’ Λ = 0.595; *p* < 0.001) and muscle strength and power variables (Wilks’ Λ = 0.567; *p* < 0.001), indicating significant overall differences between groups in the combined dependent variables.

The results of the MANCOVA analysis (Table 4) indicated significant multivariate effects of age, sex, and body height on body composition, handgrip strength, and lower limb muscle power variables (*p* < 0.01). Importantly, a significant group effect was observed for both body composition (Wilks’ Λ = 0.827; *p* = 0.005) and muscle strength and power (Wilks’ Λ = 0.691; *p* = 0.009), indicating that differences between young karate athletes of different competitive disciplines remained significant after controlling for covariates.

Regarding differences in individual parameters, ANOVA results (Table 5) showed significant differences between groups in all body composition variables (BW, TBW, PRO, MIN, and SMM; *p* < 0.001), except for PBF (*p* = 0.064), as well as in BH (*p* < 0.001). Significant differences were also observed in all absolute handgrip strength variables (FmaxR, RFDR, FmaxL, and RFDL; *p* < 0.001), as well as in relative strength variables (FmaxRRel, RFDRRel, FmaxLRel, and RFDLRel, from *p* = 0.003 to *p* = 0.041). Significant differences were further observed in CMJ height, CMJ power and CMJ Rel (*p* < 0.001).

After adjusting for sex, age, and body height, ANCOVA results (Table 6) showed that significant differences between groups remained in most body composition variables (BW, TBW, PRO, SMM, and MIN, from *p* = 0.002 to *p* = 0.045), as well as in several absolute muscle strength and muscle power variables (FmaxR, FmaxL, RFDL, and CMJ power, from *p* = 0.002 to *p* = 0.041). No significant differences were observed for PBF, relative strength and power variables, and CMJ height (*p* > 0.05). RFDR showed a tendency toward significance (F = 2.893, *p* = 0.059, η^2^ = 0.048) but did not reach statistical significance after adjustment. These results indicate that observed differences are partially influenced by age, sex and BH.

**Figure 1:**
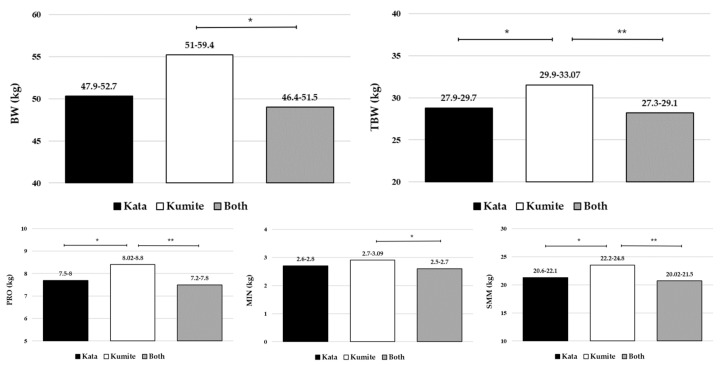
Differences in body composition variables between young karate athletes of different competitive disciplines (ANCOVA); Values are presented as mean (95% CI); *—*p* < 0.05; **—*p* < 0.01; BW—body weight; TBW—total body water; PRO—protein; MIN—minerals; SMM—skeletal muscle mass.

**Figure 2:**
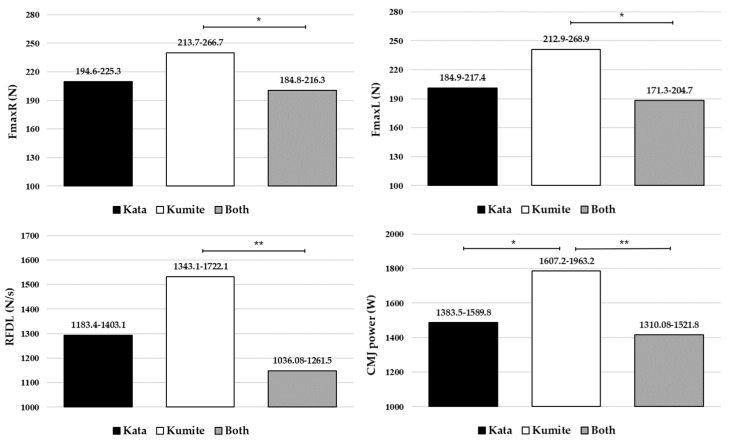
Differences in handgrip strength and lower limb muscle power parameters between young karate athletes of different competing disciplines (ANCOVA); Values are presented as mean (95% CI); *—*p* < 0.05; **—*p* < 0.01; Fmax—maximal handgrip strength; RFD—maximal handgrip rate of force development; R—right hand; L—left hand; Rel—Relative values; CMJ—countermovement jump.

Figure 1 and Figure 2 represent the results of the post hoc test (pairwise comparison, ANCOVA) for individual parameters that showed significant differences in body composition, handgrip strength, and lower limb muscle power among young karate athletes from different competitive disciplines. It can be concluded that young karate athletes specialized in kumite competition have significantly higher values of BW, TBW, PRO, MIN, SMM (body composition, *p* < 0.05) and FmaxR, Fmax L, RFDL, CMJ power (muscle strength and power, *p* < 0.05) than kata athletes and athletes specialized in both competitive disciplines. There were no significant differences between kata athletes and athletes specialized in both disciplines in any measured parameters.

## 4. Discussion

This study aimed to investigate differences in body composition, muscle strength, and muscle power among young karate athletes competing in different disciplines (kata vs. kumite vs. both). All participants were Shotokan karate practitioners, which enhances the homogeneity of the sample and supports the internal validity of the observed results. The main finding is that there were significant overall differences, as well as differences in most individual parameters (except PBF), between groups with different training and competitive backgrounds (Table 3 and Table 5). After adjustment for sex, age, and body height, most group differences remained significant, particularly for body composition and absolute muscle strength and power variables, although RFDR, CMJ height and CMJ Rel were no longer significant (Table 4 and Table 6). Post hoc analysis based on adjusted means (Figure 1 and Figure 2) indicated that kumite athletes still demonstrated higher values in most body composition, absolute handgrip strength, and lower limb muscle power variables compared to kata athletes and athletes specialized in both disciplines, while no significant differences were observed between kata athletes and those competing in both disciplines. However, some differences observed in the ANOVA analysis were reduced after adjustment, particularly for variables influenced by body size. The findings generally support the initial hypothesis, with significant between-group differences observed in most body composition, muscle strength, and power variables.

The observed differences between groups appear to be associated with competitive discipline and underlying morphological and neuromuscular characteristics, rather than exclusively reflecting discipline-specific training adaptations. After adjustment for sex, age, and body height, these differences were partially attenuated but remained significant for most variables, suggesting that anthropometric and demographic factors only partly explain the observed group differences. The results are in line with previous similar studies, which emphasised significant differences between these groups of athletes [9,10,23]. A possible explanation for these differences is a selection effect, whereby athletes with more favourable anthropometric characteristics, such as greater body height and body mass, may be more likely to be selected into kumite, which places higher demands on reach, strength, and physical contact compared to kata [9].

These differences are consistent with the nature of the movement, training, and competitive patterns specific to kata and kumite disciplines in karate. As previously mentioned, kata competition implies a standardised sequence of offensive and defensive gestures without the presence of the opponent [8]. The basic elements of proper kata technique include kime, a short isometric muscle contraction performed when a technique is concluded, expressiveness, and rhythm [10]. Opposite to kata, kumite represents the karate fight and consists of the execution of defensive and offensive techniques applied against an opponent [9]. Although kumite competitions involve noncontact fighting, the athletes must demonstrate the potential force of their movements and execute them as if they were real, using control to stop the movements so as not to inflict damage to the opponent [10]. Kumite matches last 3 min, at highly intense activities, including kicks, punches, and quick horizontal displacements [8]. From these stated facts, it is clear that kata and kumite are similar but structurally different competing and training disciplines. While kata competition and training are mainly focused on precise and fast execution of technical movements, kumite involves additional demands such as perceptual and anticipatory skills and rapid changes in direction. Both disciplines require well-developed physical capacities, including strength and appropriate body composition, although their relative importance and manifestation may differ [8,9].

It may be suggested that differences in training and competitive background are associated with higher levels of handgrip strength and lower limb muscle power in kumite athletes. Since muscle strength and power are closely related to body dimensions [27], particularly muscle mass [32], the observed differences may also be partly related to morphological characteristics, such as greater muscle mass and consequently higher body weight.

Furthermore, these relationships may be influenced by physiological and lifestyle factors, including hydration status and total body water [33], although these associations should be interpreted cautiously. Finally, previous studies have also reported a positive correlation between muscle strength and bone mineral density [34], as well as between protein intake [35], with these factors potentially being associated with higher levels of body water, protein, and mineral content in kumite athletes.

The only non-significant parameter regarding differences between groups was PBF. The percentage of body fat is considered one of the most significant parameters of body composition which influence the sport and performance success, regardless of sport categories [36,37,38], and this study confirmed this statement, since body fat percentage in these groups of subjects is similar and in a range of normal for this age category [39]. Body height, although an anthropometric measure, was included in this study as a potentially important parameter for determining differences between young karate athletes and for comparison with previous studies in combat sports. As previously stated, kumite athletes are taller than kata athletes and are specialised in both disciplines. Differences in body height may suggest that anthropometric characteristics play a role in the distribution of athletes across competitive disciplines, potentially reflecting selection processes in karate rather than training-induced adaptations. This information is leading to another aspect of view regarding differences between young karate athletes of different competing disciplines. It is assumed that kumite athletes are selected to be tall, which implies higher arm and leg span, which is important for success in competition (fight) [9]. Higher body height further implies higher body mass, muscle mass, protein, mineral and body water content, and finally muscle strength and power, as previously explained. Although kata athletes expressed lower values in all measured parameters than athletes specialised in both competing disciplines, these differences are not significant. This result is expected and logical, since both groups are practising similar movement patterns.

### Practical Applications, Limitations, and Future Research Directions

The present study provides preliminary insights into differences in body composition, muscle strength, and muscle power among young karate athletes of different competitive disciplines. These results contribute to a better understanding of the physical characteristics associated with different competitive demands in karate and may be useful for the interpretation of athlete profiles across disciplines.

However, several limitations should also be acknowledged. First, the sample was limited to children aged 10–14 years, which may restrict the generalizability of the findings. Second, although adjustments for sex, age, and body height were performed, residual confounding may still exist, which could have influenced the observed group differences.

Third, biological maturation was not assessed, which is particularly relevant in youth populations and may have affected the interpretation of the results. Fourth, the cross-sectional design does not allow for causal inferences between training background and measured variables. Additionally, this study did not include any anthropometric parameters, except for body height. Finally, body composition assessment via bioelectrical impedance analysis, the use of general rather than karate-specific physical tests, and the absence of competitive outcome measures such as weight categories, rankings, or technical scores represent further limitations of this study.

Future studies should investigate differences in a broader range of fitness parameters and morphological characteristics in larger and more diverse samples to further enhance the understanding of the specificity of different competitive disciplines in young karate athletes.

## 5. Conclusions

The study showed significant differences in most assessed parameters of body composition, muscle strength, and muscle power between young karate athletes with different training and competitive backgrounds. Kumite athletes generally demonstrated higher values of selected body composition, absolute handgrip strength, and lower limb muscle power compared to kata athletes and athletes specialized in both disciplines. No significant differences were observed between kata athletes and athletes competing in both disciplines across the measured variables. These findings suggest associations between competitive discipline and morphological and neuromuscular characteristics in young karate athletes.

## Figures and Tables

**Table 1:** Descriptive indicators of demographics and anthropometric characteristics, body composition, handgrip strength and lower limb muscle power in young karate athletes of different competitive disciplines.

		Kata		Kumite		Both	
		Mean	SD	Mean	SD	Mean	SD
Body composition	Age	11.49	1.83	12.61	0.84	11.86	1.75
	Sex (M/F)	22/26	45.8%/54.2	23/17	57.5%/42.5	24/19	55.8%/44.2%
	Training experience	3.01	0.94	3.30	0.97	3.35	1.02
	BMI (kg/m^2^)	19.79	3.73	23.24	3.72	19.90	3.06
	Training frequency	3.30	0.48	3.76	0.72	3.38	0.50
	BH (cm)	148.42	14.21	171.96	8.63	155.52	14.26
	BW (kg)	44.64	14.88	68.63	11.32	49.13	14.31
	TBW (l)	24.77	9.01	40.90	7.27	28.45	9.80
	PRO (kg)	6.64	2.46	11.00	1.99	7.61	2.67
	MIN (kg)	2.36	0.84	3.81	0.67	2.70	0.89
	SMM (kg)	18.02	7.42	31.17	6.01	20.98	8.05
	PBF (%)	23.79	9.36	18.23	11.15	21.24	9.15
Muscle strength and power	FmaxR (N)	179.36	88.71	309.30	73.39	202.79	95.02
	RFDR (N/s)	1102.20	690.10	2062.39	584.63	1305.70	684.37
	FmaxL (N)	169.24	86.79	311.91	96.76	191.00	103.59
	RFDL (N/s)	1073.76	649.35	2018.61	689.61	1169.63	649.63
	FmaxRRel (N/kg)	5.90	1.48	6.91	1.90	6.05	1.60
	RFDRRel (N/s/kg)	35.07	12.66	45.99	14.03	38.34	11.57
	FmaxLRel (N/kg)	5.55	1.55	6.82	1.82	5.63	1.86
	RFDLRel (N/s/kg)	34.39	12.90	44.14	12.88	33.98	11.38
	CMJ height (cm)	19.22	7.39	25.39	6.67	20.81	5.68
	CMJ power (w)	1133.82	949.33	2595.01	627.44	1434.22	864.01
	CMJ Rel (w/kg)	22.35	12.54	37.70	5.87	27.13	9.40

Legend: BMI—Body mass index; BH—body height; BW—body weight; TBW—total body water; PRO—protein; MIN—minerals; SMM—skeletal muscle mass; PBF—percentage of body fat; Fmax—maximal handgrip strength; RFD—maximal handgrip rate of force development; R—right hand; L—left hand; Rel—Relative values; CMJ—countermovement jump.

**Table 2:** List of variables, measurement units, and abbreviations.

No.	Variable	Measurement	Abbreviation
1.	Body height (covariate)	cm	BH
2.	Body weight	kg	BW
3.	Total body water	l	TBW
4.	Proteins	kg	PRO
5.	Minerals	kg	MIN
6.	Skeletal muscle mass	kg	SMM
7.	Percentage of body fat	%	PBF
8.	Maximal handgrip strength of the right hand	N	FmaxR
9.	Maximal rate of force development of the right hand	N/s	RFDR
10.	Maximal handgrip strength of the left hand	N	FmaxL
11.	Maximal rate of force development of the left hand	N/s	RFDL
12.	Relative handgrip strength of the right hand	N/kg	FmaxRRel
13.	Relative rate of force development of the right hand	N/s/kg	RFDRRel
14.	Relative handgrip strength of the left hand	N/kg	FmaxLRel
15.	Relative rate of force development of the left hand	N/s/kg	RFDLRel
16.	Countermovement jump height	cm	CMJ height
17.	Countermovement jump power	w	CMJ power
18.	Relative countermovement jump power	w/kg	CMJ Rel

**Table 3:** The results of MANOVA—general differences in body composition, handgrip strength, and lower limb muscle power between young karate athletes of different competing disciplines.

Wilks’ Lambda	Value	F	Sig.	Partial Eta Squared
Body composition	0.595	5.587	<0.001	0.229
Muscle strength and power	0.567	3.217	<0.001	0.247

**Table 4:** The results of MANCOVA—general differences in body composition, handgrip strength, and lower limb muscle power between young karate athletes of different competing disciplines with age, sex, and body height as covariates.

	Wilks’ Lambda	Value	F	Sig.	Partial Eta Squared
Body composition	Age	0.790	5.893	<0.001	0.210
	Sex	0.726	8.384	<0.001	0.274
	BH	0.292	53.789	<0.001	0.708
	Groups	0.827	2.210	0.005	0.091
Muscle strength and power	Age	0.721	4.103	<0.001	0.279
	Sex	0.806	2.545	0.009	0.194
	BH	0.510	10.186	<0.001	0.490
	Groups	0.691	1.937	0.009	0.169

Legend: BH—body height.

**Table 5:** The results of ANOVA—differences in individual parameters of body composition, handgrip strength and lower limb muscle power between young karate athletes of different competitive disciplines.

		df	F	Sig.	Partial Eta Squared
	BH	2	25.147	<0.001	0.299
Anthropometric and body composition	BW	2	24.018	<0.001	0.289
	TBW	2	26.176	<0.001	0.307
	PRO	2	25.695	<0.001	0.303
	MIN	2	24.786	<0.001	0.296
	SMM	2	25.703	<0.001	0.303
	PBF	2	2.818	0.064	0.046
Muscle strength and power	FmaxR	2	17.904	<0.001	0.233
	RFDR	2	16.870	<0.001	0.222
	FmaxL	2	18.950	<0.001	0.243
	RFDL	2	17.835	<0.001	0.232
	FmaxRRel	2	3.291	0.041	0.053
	RFDRRel	2	6.127	0.003	0.094
	FmaxLRel	2	4.774	0.010	0.075
	RFDLRel	2	6.001	0.003	0.092
	CMJ height	2	6.920	<0.001	0.105
	CMJ power	2	23.352	<0.001	0.284
	CMJ Rel	2	17.385	<0.001	0.228

Legend: BH—body height; BW—body weight; TBW—total body water; PRO—protein; MIN—minerals; SMM—skeletal muscle mass; PBF—percentage of body fat; Fmax—maximal handgrip strength; RFD—maximal handgrip rate of force development; R—right hand; L—left hand; Rel—Relative values; CMJ—countermovement jump.

**Table 6:** The results of ANCOVA—differences in individual parameters of body composition, handgrip strength and lower limb muscle power between young karate athletes of different competitive disciplines with age, sex, and body height as covariates.

		Adj. Mean [95% CI] Kata	Adj. Mean [95% CI] Kumite	Adj. Mean [95% CI] Both	df	F	Sig.	Partial Eta Squared
Body composition	BW	50.3 [47.9–52.7]	55.2 [51–59.4]	49 [46.4–51.5]	2	3.180	0.045	0.052
	TBW	28.8 [27.9–29.7]	31.5 [29.9–33.07]	28.2 [27.3–29.1]	2	6.622	0.002	0.103
	PRO	7.7 [7.5–8]	8.4 [8.02–8.8]	7.5 [7.2–7.8]	2	6.506	0.002	0.102
	MIN	2.7 [2.6–2.8]	2.9 [2.7–3.09]	2.6 [2.5–2.7]	2	3.665	0.029	0.060
	SMM	21.3 [20.6–22.1]	23.5 [22.2–24.8]	20.7 [20.02–21.5]	2	6.498	0.002	0.102
	PBF	22.2 [19.6–24.7]	21.3 [16.9–25.7]	21.5 [18.9–24.1]	2	0.082	0.922	0.001
Muscle strength and power	FmaxR	209.9 [194.6–225.3]	240.2 [213.7–266.7]	200.5 [184.8–216.3]	2	3.291	0.041	0.054
	RFDR	1317.1 [1199.9–1434.4]	1575.5 [1373.3–1777.7]	1291.09 [1170.7–1411.4]	2	2.893	0.059	0.048
	FmaxL	201.2 [184.9–217.4]	240.9 [212.9–268.9]	188.04 [171.3–204.7]	2	5.255	0.007	0.084
	RFDL	1293.2 [1183.4–1403.1]	1532.6 [1343.1–1722.1]	1148.8 [1036.08–1261.5]	2	6.535	0.002	0.102
	FmaxRRel	6.05 [5.6–6.4]	6.6 [5.8–7.3]	6.01 [5.5–6.4]	2	1.019	0.364	0.017
	RFDRRel	36.7 [33.7–39.8]	42.3 [37.06–47.6]	38.09 [34.9–41.2]	2	1.353	0.262	0.023
	FmaxLRel	5.8 [5.3–6.2]	6.3 [5.6–7.08]	5.5 [5.1–6.02]	2	1.584	0.209	0.027
	RFDLRel	36.3 [33.7–39.5]	39.5 [34.5–44.5]	33.5 [30.5–36.5]	2	2.580	0.080	0.043
	CMJ height	20.7 [19.3–22.1]	22.05 [19.6–24.4]	20.6 [19.1–22.03]	2	0.533	0.588	0.009
	CMJ power	1486.7 [1383.5–1589.8]	1785.2 [1607.2–1963.2]	1415 [1310.08–1521.8]	2	6.255	0.003	0.098
	CMJ Rel	26.17 [24.66–27.68]	28.87 [26.27–31.48]	26.96 [25.41–28.51]	2	1.433	0.234	0.012

Legend: BH—body height; BW—body weight; TBW—total body water; PRO—protein; MIN—minerals; SMM—skeletal muscle mass; PBF—percentage of body fat; Fmax—maximal handgrip strength; RFD—maximal handgrip rate of force development; R—right hand; L—left hand; Rel—Relative values; CMJ—countermovement jump.

## Data Availability

The raw data supporting the conclusions of this article will be made available by the authors on request.

## References

[1] Bailey R.P., Samsudin N. (2025). Martial Arts and the Problem of Definition. Philosophies.

[2] Hajder Đ., Bjelica B., Aksović N., Dobrescu T., Bubanj S. (2025). Comparative Analysis of the Effects of Training with Additional Load on Motor Fitness and Morphological Characteristics of Martial Sports Athletes. Phys. Educ. Sport. Through Centuries.

[3] Yi J., Silver D. (2015). God, Yoga, and Karate. J. Sci. Study Relig..

[4] Rutkowski T., Chwałczyńska A. (2025). The Impact of Karate and Yoga on Children’s Physical Fitness: A 10-Week Intervention Study. Appl. Sci..

[5] Chaabène H., Hachana Y., Franchini E., Mkaouer B., Chamari K. (2012). Physical and physiological profile of elite karate athletes. Sports Med..

[6] Burke D.T., Al-Adawi S., Lee Y.T., Audette J. (2007). Martial Arts as Sport and Therapy. J. Sports Med. Phys. Fit..

[7] Arsenijević R., Trikoš B., Kojić F., Toskić L., Stanković V., Aksović N., Dobrescu T. (2025). Monitoring the Subjective Internal Load in Elite Level Kumite-Karate Athletes: Role of the Rest Periods Duration, Breathing Techniques, and Training Volume. Kinesiol. Slov..

[8] Molinaro L., Taborri J., Montecchiani M., Rossi S. (2020). Assessing the Effects of Kata and Kumite Techniques on Physical Performance in Elite Karatekas. Sensors.

[9] Koropanovski N., Berjan B., Bozic P.R., Pazin N., Sanader A., Jovanovic S., Jaric S. (2011). Anthropometric and Physical Performance Profiles of Elite Karate Kumite and Kata Competitors. J. Hum. Kinet..

[10] Doria C., Veicsteinas A., Limonta E., Maggioni M.A., Aschieri P., Eusebi F., Fanò G., Pietrangelo T. (2009). Energetics of Karate (Kata and Kumite Techniques) in Top-Level Athletes. Eur. J. Appl. Physiol..

[11] Benedini S., Longo S., Caumo A., Luzi L., Invernizzi P.L. (2012). Metabolic and Hormonal Responses to a Single Session of Kumite (Free Non-Contact Fight) and Kata (Highly Ritualized Fight) in Karate Athletes. Sport. Sci. Health.

[12] Emad B., Atef O., Shams Y., El-Kerdany A., Shorim N., Nabil A., Atia A. (2020). iKarate: Karate Kata Guidance System. Procedia Comput. Sci..

[13] Gökdere F., Uylas E., Çatıkkaş F., Günay E., Ceylan H.İ., Özgören M. (2025). Integrating Kata Training into School Education: Effects on Sustained Attention and Cognitive Performance in 8–9-Year-Old Children. Children.

[14] Sastre V., Lapresa D., Arana J., Ibáñez R., Teresa Anguera M. (2021). Observational Analysis of Lateral Preference in Kumite Initiation: A Starting Point in the Longitudinal Programming of Formative Karate. Percept. Mot. Ski..

[15] Kim K., Tsuchida S., Terada T., Tsukamoto M. (2025). A Method for Detecting Preliminary Actions During an Actual Karate Kumite Match. Sensors.

[16] Kabadayı M., Karadeniz S., Yılmaz A.K., Karaduman E., Bostancı Ö., Akyildiz Z., Clemente F.M., Silva A.F. (2022). Effects of Core Training in Physical Fitness of Youth Karate Athletes: A Controlled Study Design. Int. J. Environ. Res. Public Health.

[17] Cavedon V., Milanese C., Sacristani F., Zancanaro C. (2023). Body Composition in Karate: A Dual-Energy X-ray Absorptiometry Study. Appl. Sci..

[18] Rutkowski T., Sobiech K.A., Chwałczyńska A. (2020). The Effect of 10 Weeks of Karate Training on the Weight Body Composition and FFF Index of Children at the Early School Age with Normal Weight and Overweight. Arch. Budo.

[19] Piepiora P.A. (2025). The Health Effects of Karate Training: A Review of 21st Century Research. Healthcare.

[20] Dopsaj M., Andraos Z., Richa C., Mitri A., Makdissi E., Zoghbi A., Fayyad F. (2022). Maximal and Explosive Strength Normative Data for Handgrip Test According to Gender: International Standardization Approach. Hum. Mov..

[21] Zećirović A., Ćeremidžić D., Joksimović A., Ćeremidžić T., Joksimović D., Aksović N., Toskić L., Dragoi C.-C., Ciocan V.C., Mihaela A. (2025). The Effects of Group Fitness Programs Zumba and MoFit on Body Composition Parameters in Women. Life.

[22] Jeknić V., Dopsaj M., Toskić L., Koropanovski N. (2022). Muscle Contraction Adaptations in Top-Level Karate Athletes Assessed by Tensiomyography. Int. J. Environ. Res. Public Health.

[23] Katanic B., Bjelica D., Rezic M., Selimi M., Osmani A. (2022). Differences in the Morphological Characteristics and Body Composition between Elite Montenegrin Kata and Kumite Karatekas. Sport. Mont..

[24] Toskic L., Markovic M., Simenko J., Vidic V., Cikiriz N., Dopsaj M. (2024). Analysis of body composition in men and women with diverse training profiles: A cross-sectional study. Int. J. Morphol..

[25] Marković S., Dopsaj M., Veljković V. (2020). Reliability of Sports Medical Solutions handgrip and Jamar handgrip dynamometer. Meas. Sci. Rev..

[26] Gerodimos V. (2012). Reliability of handgrip strength test in basketball players. J. Hum. Kinet..

[27] Jaric S. (2002). Muscle Strength Testing: Use of Normalisation for Body Size. Sports Med..

[28] Bagchi A., Raizada S., Thapa R.K., Stefanica V., Ceylan H.İ. (2024). Reliability and Accuracy of Portable Devices for Measuring Countermovement Jump Height in Physically Active Adults: A Comparison of Force Platforms, Contact Mats, and Video-Based Software. Life.

[29] Plakoutsis G., Zapantis D., Panagiotopoulou E.-M., Paraskevopoulos E., Moutzouri M., Koumantakis G.A., Papandreou M. (2023). Reliability and Validity of the Portable KForce Plates for Measuring Countermovement Jump (CMJ). Appl. Sci..

[30] Riggs M.P., Sheppard J.M. (2009). The relative importance of strength and power qualities to vertical jump height of elite beach volleyball players during the counter-movement and squat jump. J. Hum. Sport. Exerc..

[31] Pallant J. (2011). SPSS Survival Manual: A Step by Step Guide to Data Analysis Using the SPSS Program.

[32] Chen L., Nelson D.R., Zhao Y., Cui Z., Johnston J.A. (2013). Relationship between Muscle Mass and Muscle Strength, and the Impact of Comorbidities: A Population-Based, Cross-Sectional Study of Older Adults in the United States. BMC Geriatr..

[33] Silva A.M., Fields D.A., Heymsfield S.B., Sardinha L.B. (2011). Relationship between Changes in Total-Body Water and Fluid Distribution with Maximal Forearm Strength in Elite Judo Athletes. J. Strength Cond. Res..

[34] Pelegrini A., Bim M.A., Alves A.D., Scarabelot K.S., Claumann G.S., Fernandes R.A., de Araújo Pinto A. (2022). Relationship between Muscle Strength, Body Composition and Bone Mineral Density in Adolescents. J. Clin. Densitom..

[35] Sahni S., Mangano K.M., Hannan M.T., Kiel D.P., McLean R.R. (2015). Higher Protein Intake Is Associated with Higher Lean Mass and Quadriceps Muscle Strength in Adult Men and Women. J. Nutr..

[36] Aikawa Y., Murata M., Omi N. (2020). Relationship of Height, Body Mass, Muscle Mass, Fat Mass, and the Percentage of Fat with Athletic Performance in Male Japanese College Sprinters, Distance Athletes, Jumpers, Throwers, and Decathletes. J. Phys. Fit. Sports Med..

[37] Högström G.M., Pietilä T., Nordström P., Nordström A. (2012). Body Composition and Performance: Influence of Sport and Gender among Adolescents. J. Strength Cond. Res..

[38] Fields J.B., Merrigan J.J., White J.B., Jones M.T. (2018). Body Composition Variables by Sport and Sport-Position in Elite Collegiate Athletes. J. Strength. Cond. Res..

[39] Stupnicki R., Tomaszewski P., Milde K., Czeczelewski J., Lichota M., Głogowska J. (2009). Body Fat-Based Weight Norms for Children and Youths. Pediatr. Endocrinol. Diabetes Metab..

